# MicroRNA expression in human endometrial adenocarcinoma

**DOI:** 10.1186/s12935-014-0088-6

**Published:** 2014-11-14

**Authors:** Sanja Jurcevic, Björn Olsson, Karin Klinga-Levan

**Affiliations:** Systems Biology Research Centre - Tumor Biology, Bio Science, University of Skövde, SE541 28, Skövde, Sweden; Systems Biology Research Centre - Bioinformatics, Bio Science, University of Skövde, Skövde, Sweden

**Keywords:** Endometrial adenocarcinoma, MicroRNA, Quantitative polymerase chain reaction

## Abstract

**Background:**

MicroRNAs are small non-coding RNAs that play crucial roles in the pathogenesis of different cancer types. The aim of this study was to identify miRNAs that are differentially expressed in endometrial adenocarcinoma compared to healthy endometrium. These miRNAs can potentially be used to develop a panel for classification and prognosis in order to better predict the progression of the disease and facilitate the choice of treatment strategy.

**Methods:**

Formalin fixed paraffin embedded endometrial tissue samples were collected from the Örebro university hospital. QPCR was used to quantify the expression levels of 742 miRNAs in 30 malignant and 20 normal endometrium samples. After normalization of the qPCR data, miRNAs differing significantly in expression between normal and cancer samples were identified, and hierarchical clustering analysis was used to identify groups of miRNAs with coordinated expression profiles.

**Results:**

In comparisons between endometrial adenocarcinoma and normal endometrium samples 138 miRNAs were found to be significantly differentially expressed (p < 0.001) among which 112 miRNAs have not been previous reported for endometrial adenocarcinoma.

**Conclusion:**

Our study shows that several miRNAs are differentially expressed in endometrial adenocarcinoma. These identified miRNA hold great potential as target for classification and prognosis of this disease. Further analysis of the differentially expressed miRNA and their target genes will help to derive new biomarkers that can be used for classification and prognosis of endometrial adenocarcinoma.

**Electronic supplementary material:**

The online version of this article (doi:10.1186/s12935-014-0088-6) contains supplementary material, which is available to authorized users.

## Background

Endometrial cancer is the most common malignancy in the female population in developed countries. According to the European Network of Cancer Registries, 82,530 endometrial cancer cases were recorded in 2008 in Europe [1]. Endometrial cancer is classified as endometrioid adenocarcinoma (type I) and serous carcinoma (type II). The most dominant subtype, type I, occurs in pre- and post-menopausal women and is frequently developed from endometrial hyperplasia. It is related to estrogen stimulation and has a good prognosis. Type II, which develops from atrophic endometrium, occurs mainly in postmenopausal women, is estrogen independent and has poor prognosis [2,3]. Five-year survival from endometrioid adenocarcinoma (EAC) is 80% among early diagnosed cases [4]. However, for patients with advanced-stage or recurrent endometrioid adenocarcinoma, prognosis is very poor and therefore novel biomarkers for early detection and outcome prediction could reduce the mortality.

MicroRNAs (miRNAs) are small RNA molecules that regulate gene expression in two main ways: by degradation of their target mRNAs or by promoting translational repression [5]. The insight that miRNAs play crucial roles in biological processes including cellular differentiation, proliferation and apoptosis, indicate that abnormal expression of miRNAs can contribute to the development of human cancer [6]. Aberrant miRNA expression has been reported in several human cancers. For example, down-regulation of miR-143 and miR-145 has been reported in colorectal cancer [7], and down-regulation of miR-15 and miR-16 in chronic lymphatic leukemia [8], while increased expression of members of the miR-17-92 cluster has been reported in lung cancer [9] as well as in diffuse B-cell lymphomas [10].

Formalin-fixed paraffin-embedded (FFPE) tissues are routinely archived in most hospitals, and this material is widely used for discovery of clinically useful biomarkers [11]. Gene expression analysis of RNA isolated from FFPE tissues is challenging due to RNA degradation during fixation and storage as well immediately after resection of tumors [12]. Previous studies indicate that miRNAs may be less affected by formalin fixation and paraffin embedding than mRNA, which is probably due to their smaller size and thereby slower degradation [13,14]. These features make miRNAs particularly attractive as biomarkers for cancer diagnosis and prognosis [15].

In this study, we have investigated the expression of 742 miRNAs in human endometrioid adenocarcinoma and normal samples from the endometrium by using real-time quantitative PCR to identify miRNAs that could serve as diagnostic and prognostic markers for this type of cancer.

## Results and discussion

### MiRNAs differentially expressed in endometrial cancer and normal endometrial tissue

To date, there are six studies of global miRNA expression in endometrioid adenocarcinoma [16-21]. These studies have utilized microarray and/or qPCR methods assessing from 157 to 866 miRNAs in cohorts ranging from twenty to over one hundred samples. Altogether, 21 miRNAs were found to be up-regulated in EAC compared to normal endometrium common to at least two of the six studies. The studies included a variety of histological subtypes, which could explain the low number of common differentially expressed miRNAs [16-21].

**Figure 1:**
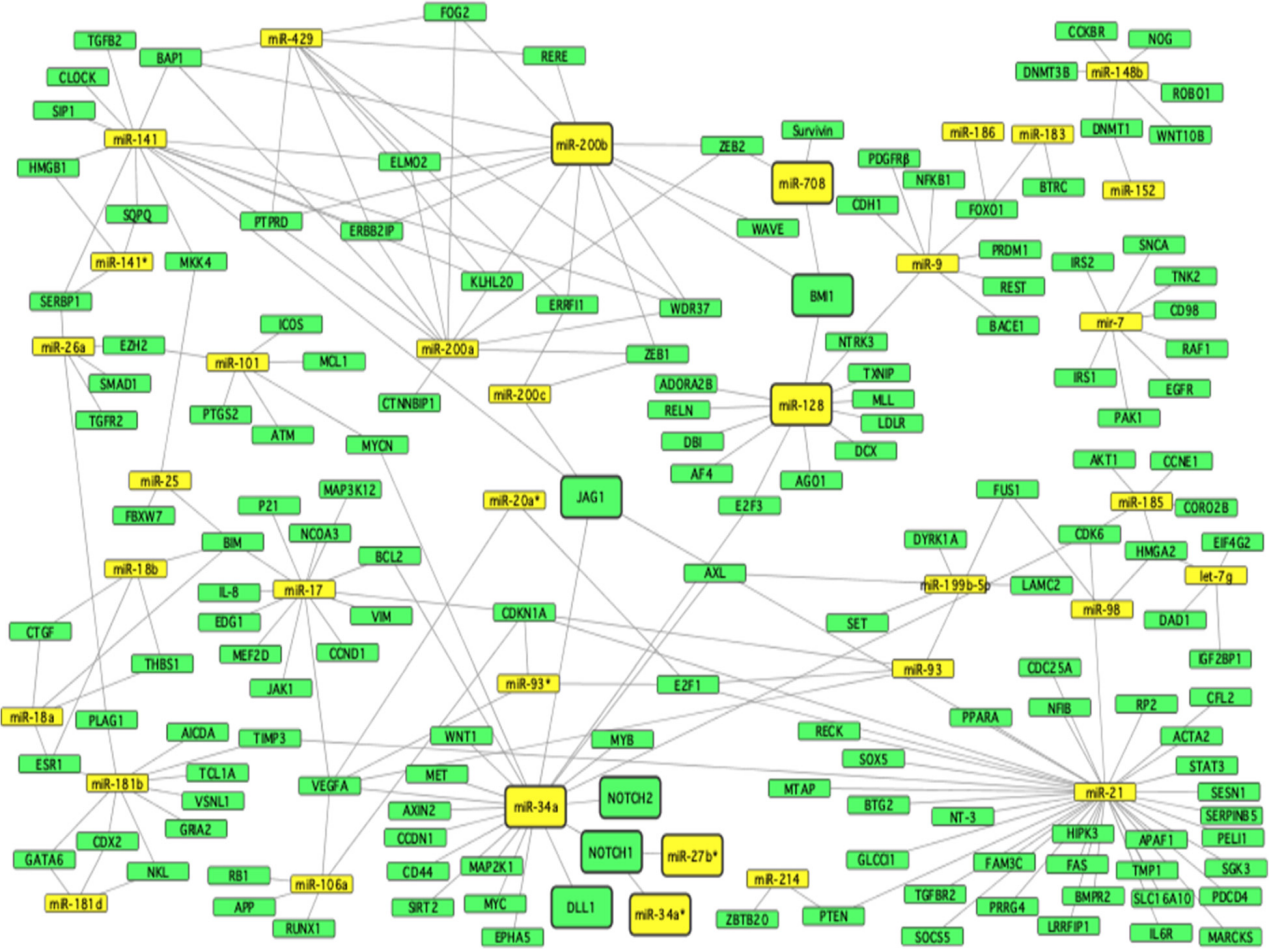
**Network of miRNAs (yellow) and their target genes (green).** Larger boxes represent miRNAs and target genes discussed in the text.

In the present study a large-scale miRNA expression analysis of 742 miRNAs was performed on 50 samples, comprising 30 cancer and 20 normal endometrium samples (see Material and methods). In order to determine the miRNA expression, we used a real-time PCR assay system based on LNA probes. Comparison between cancer and normal endometrium samples revealed that 138 miRNAs were significantly differentially expressed (p < 0.001), where 128 miRNAs were up-regulated and 10 were down-regulated (Additional file 1: Table S1). The large of number differentially expressed miRNA may seem high, but as can be seen in the network image (Figure 1), the differentially expressed genes identified herein are involved in pathways that are often deregulated in cancer. Among the top differentially expressed miRNAs, miR-183 and miR-182 are most up-regulated in cancer samples (highest fold change) while miR-1247 and miR-199b-5p were most down-regulated in cancer samples compared to normal samples (Table 1).

**Table 1:** **List of the most differentially expressed miRNAs in endometrial cancer**

**miRNA**	**Fold change**	**p-value**
**Up-regulated**		
miR-183	39.68	4.28 × 10^−15^
miR-182	30.55	2.89 × 10^−14^
miR-429	19.54	7.66 × 10^−12^
miR-135a	16.58	4.79 × 10^−10^
miR-9-3p	16.55	1.77 × 10^−10^
miR-9	16.49	4.43 × 10^−07^
miR-135b	15.95	6.10 × 10^−08^
miR-200a-5p	15.92	4.72 × 10^−13^
miR-218	15.29	1.65 × 10^−10^
miR-18a-3p	15.04	1.03 × 10^−11^
**Down-regulated**		
miR-1247	−5.72	2.07 × 10^−06^
miR-199b-5p	−5.22	3.42 × 10^−06^
miR-214	−4.80	8.39 × 10^−07^
miR-370	−4.39	6.70 × 10^−08^
miR-424-3p	−3.90	1.57 × 10^−07^
miR-376c	−3.68	2.77 × 10^−05^
miR-542-5p	−3.59	1.58 × 10^−05^
miR-758	−2.57	8.54 × 10^−06^
miR-377	−2.53	1.07 × 10^−05^
miR-337-5p	−2.19	6.91 × 10^−05^

In the former studies, four miRNAs (miR-182, miR-183, miR-200a and miR-200c) were found to be up-regulated in four of the six surveys. Among the 21 miRNAs differentially expressed in the previous studies, 12 were also found to be dysregulated in our set of EAC (Additional file 2: Table S2).

Of the 138 miRNAs that were identified in our study, 112 were not included in any of the previous reports of miRNA expression studies in endometrial adenocarcinoma. One example is miR-181b, which in the present study was shown for the first time to be up-regulated (fold change 4.11) in EAC. A validated target gene of miR-181b is *TIMP3*, tissue inhibitor of metalloproteinases-3, which is a tumor suppressor gene that has been reported as down-regulated in EAC [22]. Up-regulation of miR-181b leads to lower expression of *TIMP3* [23], which indicates that miR-181b has an oncogenic function. Furthermore, among the miRNAs not recognize before, we found that the expression of miR-148b and miR-335 were statistically significantly higher in cancer samples compared to normal endometrium. These two miRNAs regulate genes (*WNT10B* and *SOX4*, respectively) that are members of the *Wnt* signaling pathway. The Wnt signaling pathway has been studied in recent years because many of its members play significant roles in tumor development. *SOX4* is up-regulated in many cancers and seems to act as an oncogene, which enhances β-catenin/TCF activity [24]. Previous studies have shown that *WNT10B* was absent in normal endometrial cells but expressed in endometrial cancer cells [25]. It can be implied here that *WNT10B* is important for β-catenin/TCF activity.

**Figure 2:**
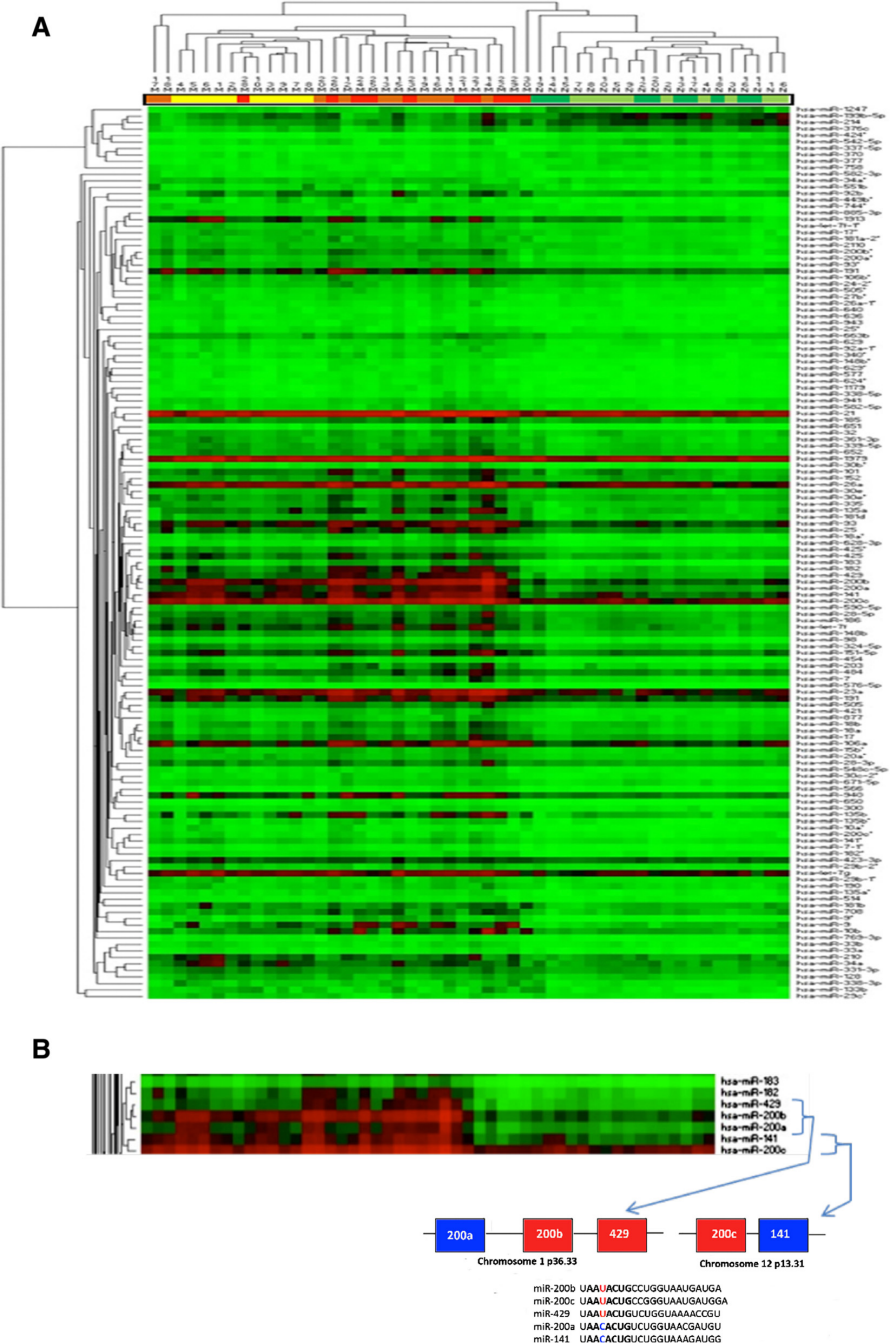
**Hierarchical clustering of endometrial cancer versus normal endometrium. A)** The color scale in the heatmap reflects expression levels of each miRNA in each sample (*red*: high expression, *green*: low expression). Samples are colored according to class (*light green*: proliferative phase, *dark green*: secretory phase, *yellow*: FIGO I. *orange*: FIGO II, *red*: FIGO III). **B)** Members of the miR-200 family and their chromosomal location.

Hierarchical clustering of the 138 differentially expressed miRNAs showed a clear distinction between normal and cancer tissues (Figure 2A). As shown in the figure at the top, endometrial samples are grouped into one cancer cluster and, one cluster with normal samples with one exception; one cancer samples (30 M) cluster among the normal samples. Two clusters of miRNAs could be identified, where the first cluster includes 10 down regulated miRNAs. Six of these miRNAs are located on chromosome 14, but still no common target genes have been identified among these miRNAs. Moreover, according to the miRBase database (www.mirbase.org) these miRNAs do not belong to the same family or cluster.

The second cluster includes miRNAs that regulate genes involved in several pathways that often are altered in endometrial adenocarcinoma. For example, miRNA-17 and miRNA-34a regulate two genes, *BCL2* and *CCND1*, which are involved in the PI3K/ Akt signaling pathway [26,27], which often is altered in EAC and involved in the development of the disease. Several miRNAs in the second cluster have common target genes. For example, *E2F3* (a target of miRNA-34a and miRNA-128) is involved in cell cycle regulation and the *p53*-signaling pathway.

### Correlation between altered miRNA expression and pathological characteristics of endometrial adenocarcinoma

**Figure 3:**
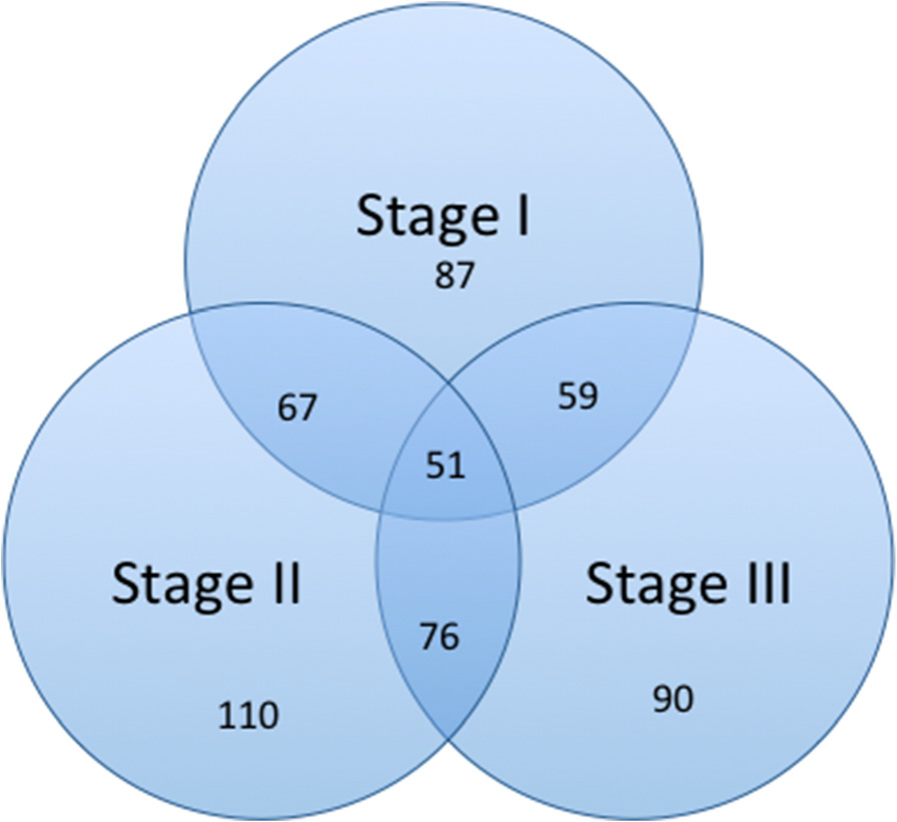
**Venn diagram summarizing differentially expressed miRNAs between the stages.**

When comparing each FIGO stage with normal endometrium we found 87 miRNAs to be differentially expressed in FIGO stage I (8 down- and 79 up-regulated), 110 miRNAs in FIGO stage II (3 down- and 107 up-regulated), and 90 miRNAs in FIGO stage III (5 down- and 85 up-regulated) (p < 0.001). Among these miRNAs, 51 (37%) were differentially expressed in all three stages (Figure 3), suggesting that deregulation of these miRNAs are early events in tumor development. Analysis of the qPCR data showed that all members of the miR-200 family (miR-200a, miR- 200b, miR-200c, miR-141 and miR-429) exhibit strongly correlated expression patterns (Figure 2B) and up-regulated in all stages of EAC compared to normal endometrium, which confirms results reported in previous studies. Gregory et al. reported evidence that suggests a crucial role for the miR-200 family members in regulation of *ZEB1* and *ZEB2* genes and in the induction of epithelial to mesenchymal transition (EMT) in several carcinoma types [28]. Furthermore, an inhibition of the miR-200 family using anti-miRs resulted in reduction of cell proliferation and enhanced the cytotoxic effect in endometrial cancer HEC-1A and Ishikawa cell lines [29].

One of the miRNAs that is differentially expressed in FIGO stage I is mir-214, which regulates expression of *PTEN*, which is a tumor suppressor gene that produces a protein with tyrosine kinase function. In endometrial adenocarcinoma, mutation in *PTEN* has been identified in up to 86% of EACs with microsatellite instability [4]. The presence of *PTEN* mutations in endometrial hyperplasia is well documented, suggesting that it is an early event in carcinogenesis. Most miRNAs that regulate genes involved in the PI3K/Akt and MAPK pathways show aberrant expression in early stage endometrial cancer.

The aberrant expression of miR-18a* was correlated with FIGO stage II. *KRAS* is a target of miR-18a* and *KRAS* mutations are detected in approximately 10% to 30% of endometrial adenocarcinomas [30]. During tumor development, activated RAS proteins facilitate cellular proliferation as well as cellular growth and also enhance cell survival. Since *KRAS* mutations like *PTEN* mutations, are found in endometrial hyperplasia at a similar rate in EAC suggests that mutation is an early event in carcinogenesis. By targeting *KRAS*, miR-18a* represses proliferation and growth of cancer cells [31]. Taken together, these data suggest that miR-18a* may serve as a potential target for endometrial cancer treatment.

### Validated targets of deregulated miRNAs

Experimentally validated target genes of the deregulated miRNAs were extracted from miRecord (http://mirecords.biolead.org). We then obtained 216 experimentally validated target genes for 67 of the differentially expressed miRNAs in the present study. Subsequently, KEGG pathway analysis of these target genes was performed using DAVID [32], which revealed several pathways relating to cancer. A total of 44 pathways, involving 106 target genes, were collected from KEGG (Table 2). Thirteen of the 44 pathways are often disrupted in different cancer types: melanoma, pancreatic cancer, bladder cancer, prostate cancer, colorectal cancer, glioma, non-small cell lung cancer, small cell lung cancer, acute myeloid leukemia, renal cell carcinoma, thyroid cancer, basal cell carcinoma and chronic myeloid leukemia.

**Table 2:** **Top five pathways associated to differentially expressed miRNAs in EAC**

**Term**	**Description**	**Number of target genes (%)**	**P value**
KEGG:04010	MAPK signaling pathway	14 (7.3%)	<0.05
KEGG:04115	p53 signaling pathway	10 (5.2%)	<0.05
KEGG:04012	ErbB signaling pathway	10 (5.2%)	<0.05
KEGG:04630	Jak-STAT signaling pathway	9 (4.7%)	<0.05
KEGG:04310	Wnt signaling pathway	8 (4.1%)	<0.05

The MAPK signaling pathway was enriched (p < 0.05), and 14 genes were targets of the differentially expressed miRNAs in this study (Table 2). The *MAPK* signaling pathway plays essential role in several cellular processes such as proliferation, differentiation and development. MAPKs genes are coding for the primary end points for a pathway included activation of MAPKKK, which in turn phosphorylate MAPKK, that phosphorylate the MAPK kinase itself [33]. Deregulation of *MAPK* signaling pathway has been shown to be associated with several diseases including various types of cancers [34]. Another important pathway, WNT signaling pathway, was also enriched (p < 0.05). Members of the Wnt family participate in multiple biological processes such as cell growth and differentiation during embryonic development [35]. Several genes including oncogene *MYC*, tumor suppressor gene *APC* and negative regulator of WNT pathway *AXIN2* were regulated by miR-34a, miR-135a and miR-135b.

Regulatory networks of differentially expressed miRNAs and target genes identified in our study were visualized with the aid of Cytoscape software (http://cytoscape.org/). The network includes all miRNAs that have two or more validated targets (35 of 67) and a total of 153 target genes (Figure 1). One of the target genes in the network, *NOTCH1* is regulated by miR-34a, miR-34a* and miR-27b*. The *Notch* signaling pathway plays an important role in cellular proliferation, differentiation and apoptosis. The Notch family consists of four receptors (NOTCH1-4) and corresponding ligands (DLL1, DLL2, DLL4, JAG 1 and JAG *2*), which are all up-regulated in endometrial cancer. It was also shown that the expression of the corresponding members of the gene family were up-regulated in higher FIGO stages and in poorly differentiated tumors [36]. Moreover, miR-34a regulates the expression of other members of the Notch family (*NOTCH2, JAG 1* and *DLL2*). Another highlighted gene in the network, *BMI1* is regulated by miR-128, miR-200b and miR-708. The gene is a member of the polycomb group (PcG) of genes that are involved in transcriptional regulation by remodeling chromatin. Low expression of *BMI1* is associated with vascular invasion and loss of hormone receptors in endometrial cancer [37].

## Conclusion

We have identified 138 miRNAs that differentially expressed between normal and malignant tissues. Hierarchical clustering revealed that the samples were in principle classified according to the feature of the samples (malignant or normal). Certain miRNAs are differentially expressed between FIGO stages, which indicate that miRNAs can be used to discriminate between early and advanced tumors. In addition, we have identified aberrant miRNAs that have not previously been described in connection with EAC. Some of these miRNAs are involved in pathways, which often are altered in EAC and contribute to the development of the disease.

Further analysis of these miRNAs and their target genes will help to derive new biomarkers that can be used for classification and prognosis of endometrial adenocarcinoma.

## Methods

### Patient material

Endometrial tissue samples were obtained from 50 patients from the University Hospital of Örebro. The specimens included 30 EAC and 20 normal endometrium samples. Ten of the normal endometrial samples were obtained from the proliferative phase and 10 from the secretory phase. The staging for all patients was decided according to the International Federation of Gynecology and Obstetrics (FIGO) classification system. Ten of the malignant samples where of FIGO stage I, 10 of stage II and 10 of stage III. Obtained tissues were formalin fixed and paraffin embedded. The 20 samples from normal endometrium were collected from women who had undergone hysterectomy for nonmalignant conditions. The study was approved by the Regional Ethical Committee Uppsala-Örebro (Number 2011/123).

### RNA isolation and quantitative real-time PCR

A pathologist marked normal and malignant tissue areas on the formalin-fixed paraffin-embedded tissue blocks. Subsequently, three 0.6 mm cores were punched out using the Tissue Micro Array equipment (Pathology devices, Westminster, USA). Total RNA was isolated from the tissues using a Recover All Total Nucleic Acid Isolation Kit optimized for FFPE samples (Ambion, Foster City, CA, USA) according to the manufacturer’s protocol. Quality and quantity of the RNA samples were determined in a NanoDrop ND-1000 Spectrophotometer (NanoDrop Technologies, USA). Synthesis of cDNA was performed using the Universal cDNA synthesis kit (Exiqon, Denmark), according to the manufacturer’s instructions. In brief, a poly-A tail was added to the 3′ end of the RNA and then cDNA was synthesized using a poly (T) primer with a 3′ degenerate anchor and a 5′ universal tag. Synthetic RNA spike-in was added to all total RNA samples prior to labeling and later used for quality control. Expression profiling was performed using the miRCURY LNA™ Universal real time microRNA polymerase chain reaction system, Ready-to-use Human Panel I and II (Exiqon) including 742 miRNAs, six endogenous control genes, an inter-plate calibrator in triplicates and a primer set for detection of a synthetic RNA spike-in (provided in the Universal cDNA synthesis kit). All reactions were performed in a LightCycler 480 real-time PCR system (Roche) in 384 well plates.

### Data analysis

All normalization and statistical analyses of qPCR data were performed in the software GenEx (MultiD Analyses AB, Göteborg, Sweden). The first step in the analysis was to compensate for run-to-run differences by normalization with interplate calibrators. The second step was to correct for the reaction efficiencies using RNA spike in. The third step included identification of the most stable endogenous control genes by GeNorm and NormFinder, which were used for the subsequent normalization (Additional files 3 and 4).

We used the two-sided Student’s t-test with a stringent p-value threshold (p < 0.001) to identify miRNAs with differential expression levels between normal endometrium and endometrial adenocarcinoma. Hierarchical clustering of the differentially expressed miRNAs was performed in the PermutMatrix software [38], using Pearson correlation and average linkage. Moreover, we have performed pathways analysis on validated target genes of the differentially expressed miRNAs based on the KEGG database. To illustrate the impact of miRNA regulation, the Software Cytoscape was used to create a network, where the differentially expressed miRNAs and their target genes were included.

## Additional files

Additional file 1: Table S1. MiRNA differentially expressed between endometrial adenocarcinoma and normal endometrium. Additional file 2: Table S2. Differentially expressed microRNAs in endometrial carcinoma compared with normal tissue in least two of the six current studies of miRNA expression in EC. Additional file 3: Table S3. Normalized expression values. Additional file 4: Table S4. Raw data from QPCR.

## Abbreviations

miRNA: microRNA
qPCR: Quantitative real-time PCR
EAC: Endometrial adenocarcinoma
FFPE: Formalin-fixed paraffin-embedded tissues
FIGO: The International Federation of Gynecology and Obstetrics
